# Improving the Forming Quality of Laser Dynamic Flexible Micropunching by Laser Pre-Shocking

**DOI:** 10.3390/ma13173667

**Published:** 2020-08-19

**Authors:** Jindian Zhang, Zongbao Shen, Youyu Lin, Kai Liu, Guoyang Zhou, Yang Wang, Lei Zhang, Pin Li, Huixia Liu, Xiao Wang

**Affiliations:** 1 School of Mechanical Engineering, Jiangsu University, Zhenjiang 212013, China; Zjd965138386@163.com (J.Z.); ZZZLlei@163.com (L.Z.); lip@ujs.edu.cn (P.L.); lhx@ujs.edu.cn (H.L.); wx@ujs.edu.cn (X.W.); 2 School of Materials Science & Engineering, Jiangsu University, Zhenjiang 212013, China; 3 SUMEC Hardware & Tools Co., Ltd., Nanjing 210032, China; linyouyu@sumec.com.cn (Y.L.); kl@sumec.com.cn (K.L.); zhougy@sumec.com.cn (G.Z.); wangyang@sumec.com.cn (Y.W.)

**Keywords:** laser pre-shocking, grain refinement, laser dynamic flexible micropunching, forming quality

## Abstract

Laser pre-shocking (LPS) was introduced into the laser dynamic flexible micropunching process to refine the grain size of a workpiece to improve the forming quality of punched parts. T2 copper foils with five different grain sizes and seven different laser power densities with and without LPS were used for the experiment. The results showed that the grains are refined and the average surface roughness $R_{a}$ decreases after LPS. For copper foils annealed at 650 °C, the value of $R_{a}$ decreases from 0.430 to 0.363 µm. The increase in laser energy density and grain size leads to the deterioration of the fracture surface. LPS can improve the quality of the fracture surface. Compared with punched holes without LPS, the dimensional accuracy and shape accuracy of punched holes can be improved by LPS. When grain size is close to the thickness of the copper foil, the forming quality of the punched parts becomes uncertain, owing to the difference in the orientation of the initial grains. The instability of laser dynamic flexible micropunching can be reduced by LPS. Especially, the improvement of forming quality of the punched part brought by LPS is significant for the copper foils with coarse grains.

## 1. Introduction

Microelectromechanical systems (MEMS) are becoming increasingly important and widely used in the industry, owing to the growing demand for parts with microsize features. The miniaturization of traditional macroplastic forming processes to meet demands for the large-scale, high-efficiency, and short-cycle machining of microparts is also becoming a research hotspot [1]. Micromanufacturing methods, such as microdrawing [2], microforming [3], and micropunching [4], have emerged one after the other and play important roles in actual production. However, numerous problems exist in the traditional microforming process, such as low processing efficiency, microforming device processing difficulties, and alignment accuracy control between the female die and male die. Such problems can generate considerable limitations in the process of industrialization and industrial production. As a representative of the new microforming process, laser shock microforming has garnered considerable attention. Laser shock microforming technology [5], as a high strain rate forming technology, is characterized by high-amplitude shock wave pressure generated by the interaction of a pulsed laser and a material to achieve the plastic deformation of the material. The advantages of noncontact forming, controllable energy, and half-mold molding technology indicate that laser shock microforming technology is highly suitable for microparts production and can avoid the problem of mold assembly and alignment effectively.

Laser shock micropunching is a type of laser shock microforming technology that can solve current problems, such as difficulties in producing complex punched parts and controlling punching clearance. Liu et al. [6] used laser shock waves as micro punches to punch 10 µm diameter microholes on 250 µm diameter metal foils and found that the cross-sectional quality of the laser-punched holes was satisfactory. However, direct laser shocking on the surface of a workpiece affects the surface morphology of the workpiece, and the laser energy is a Gaussian distribution, which also affects the forming quality of the punched parts. To overcome these deficiencies, Liu et al. [7,8,9] proposed a combination method of laser shock processing and rubber forming processing and applied it to micropunching and microbulging processes. The results showed that the combination method can attain improved forming quality. The presence of a plasticine medium has been proven to improve forming precision effectively by reducing the rebound effect, and the rubber comes in close contact with the workpiece during forming, thereby indirectly increasing the stiffness of the sample and prolonging the loading duration [10].

During the microforming process, the grain size of a workpiece has a substantial influence on the forming quality of microformed parts, which is different from macro plastic forming. Owing to the miniaturization of the feature size of microparts, the influence of the size effect is an important factor that cannot be ignored [11]. Xu et al. [12] conducted microcompression testing on pure Al with different grain and geometric sizes under different strain rates and deformation temperatures. The authors suggested that the flow stress of materials with different grain sizes decreases as geometric size decreases. The surface roughness and surface quality of an ultrafine-grained pure Al are substantially better than those of a coarse-grained pure Al. Through a unidirectional axial tensile test of pure aluminum, Janssen et al. [13] found that the greater the ratio of foil thickness to initial grain size, the stronger the flow stress of the material. Zheng et al. [14] investigated the effect of initial grain size and laser power density in laser shock bulging of copper foil and found that the microhardness in the laser-shocked region increases and the surface roughness of the bulged part increases with the increase in the grain size. Raulea et al. [15] investigated the effect of grain size on plastic deformation through unidirectional axial microdrawing, microbending, and micropunching experiments and found that in the micropunching experiments, grain orientation has a significant effect on changes in material punching performance when only one grain exists in the thickness direction. Based on the above research, under quasistatic loading conditions, grain size has been proven to be an important factor that affects punching quality during micropunching. In addition, the impact of the size effect on the laser shock micropunching process is worthy of attention. In recent years, many studies focused on the impact of grain size on the quality of laser shock micropunching. Fenske et al. [16] found that the microstructure of metal foils has a considerable impact on the forming quality of the laser shock micropunching process. When the average grain size of a metal foil is greater than its thickness, increased and inhomogeneous burr formations can be observed, and a high risk of incomplete cutting exists, owing to premature rupture within the pressurized area. Zheng et al. [17] studied the laser micropunching experiments of copper foils with different initial grain sizes under different laser power densities. The study results indicated that the shape accuracy of the punching holes decreases as the initial grain size increases. When the foil thickness nears the initial grain size, single crystal shear deformation may occur in the laser shock micropunching process, and the mechanical properties of the single grain dominate the forming quality of the punched parts.

In summary, grain size is an important factor in the forming quality of punched parts in terms of punching accuracy and surface quality. Moreover, deformation induced by shock loading has an effect on surface finish. As laser energy increases, the average surface roughness ($R_{a}$) in the forming region increases rapidly, owing to surface free forming [18]. To solve this problem, laser pre-shocking (LPS) is applied to the laser dynamic flexible micropunching process in this study. Through grain refinement, the plastic deformation ability of a workpiece is improved after LPS, thereby ensuring the precision and surface quality of the laser-micropunched parts. Laser shocking has been proven to be an effective technique for grain refinement. Lu et al. [19] studied the grain refinement of ANSI 304 stainless steel under multiple laser shot peening. Through experimental observation and analysis, a grain refinement mechanism of the face-centered cubic materials with very low stacking fault energy under laser shock peening is obtained. Zhang et al. [20] investigated the high spatial resolution characterization of laser shock-treated copper thin films and found that nanohardness in the shocked region increased more than 11%. Luo et al. [21] investigated the effect of laser shock peening on elastic modulus and nanohardness of the LY2 aluminum alloy. The results showed that the values of nanohardness in the laser-shocked region were increased by 58.13%, due mainly to the grain refinement. Dai et al. [22] analyzed the effect of laser wave planishing processing on the surface roughness of the LY2 aluminum alloy. The results indicated that the laser shock could reduce the surface roughness of the LY2 aluminum alloy from 1.88 to 0.59 µm. Yang et al. [23] proposed a laser shock flattening method to manufacture copper foil with high performance. The experimental results showed that the surface roughness decreased by 67.0%, from 52.1 to 17.2 nm. Man et al. [24] investigated the application of LPS technology to reduce surface roughness in the laser shock micropattern process. In this paper, grain-refined workpieces are obtained by LPS, and laser dynamic flexible micropunching experiments are performed under seven laser energy power densities and four different grain sizes. Evaluation is performed on the punched parts of copper foils with and without LPS for shape accuracy, dimensional accuracy, fracture morphology, and microstructure to analyze the effect of LPS on forming quality in laser dynamic flexible micropunching.

## 2. Experiments

### 2.1. Experimental Setup and Method

**Figure 1:**
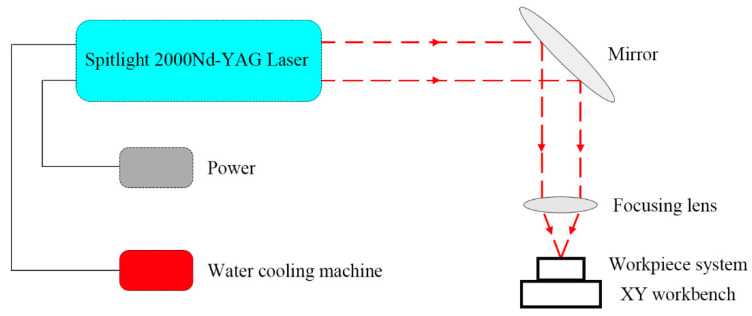
Schematic of the laser dynamic flexible micropunching process.

**Figure 2:**
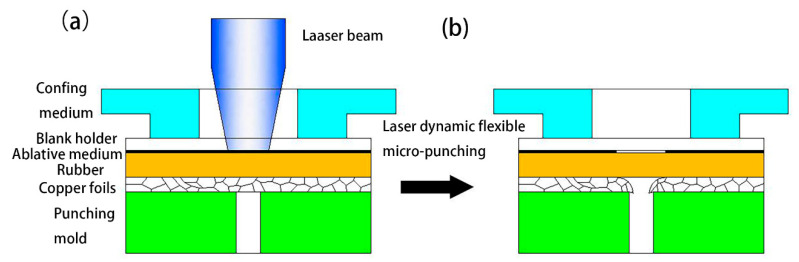
Process of laser dynamic flexible micropunching without laser pre-shocking (LPS). (**a**) Before laser dynamic flexible micropunching; (**b**) After laser dynamic flexible micropunching.

**Figure 3:**
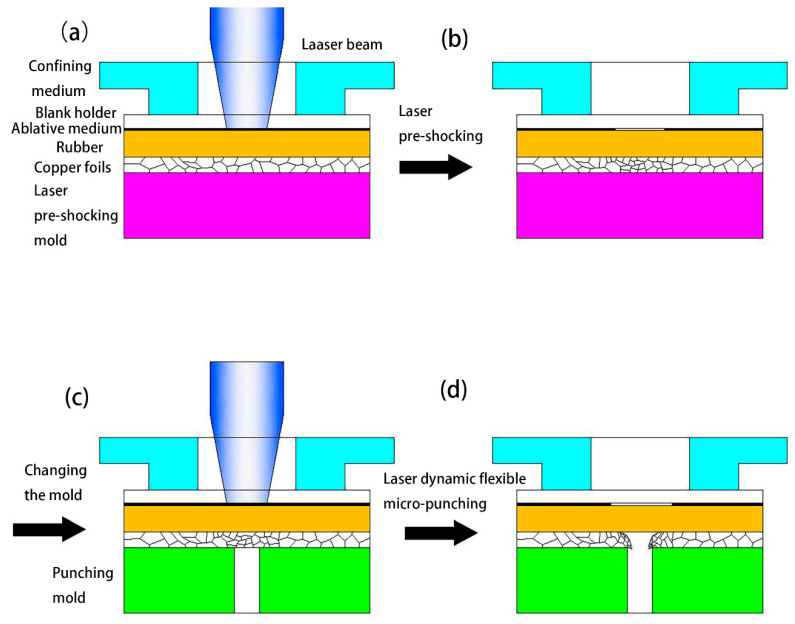
Process of laser dynamic flexible micropunching with LPS. (**a**) Before LPS; (**b**) After LPS; (**c**) Before laser dynamic flexible micropunching; (**d**) After laser dynamic flexible micropunching.

Figure 1 shows a schematic of the laser dynamic flexible micropunching process. The forming system consists of power, a water cooling machine, a Spitlight 2000 Nd-YAG laser, a mirror, a focusing lens, a workpiece system, and an XY workbench. In addition, the workpiece system consists of confining medium, blank holder, ablative medium, rubber, copper foil, and mold. The laser dynamic flexible micropunching process without LPS is shown in Figure 2, and the laser dynamic flexible micropunching process with LPS is shown in Figure 3. An ablative medium absorbs the laser pulse under the action of a blank holder and confining medium, thereby quickly vaporizing into a high-temperature and high-pressure plasma. The plasma absorbs the laser energy continuously to form a shock wave and acts on the rubber. As shown in Figure 3a,b, during LPS, a workpiece is subjected to the laser shock wave pressure indirectly transmitted by the rubber and the supporting force of a mold, and the grains inside the workpiece are refined. In the laser dynamic flexible micropunching stage, as shown in Figure 3c,d, the workpiece is subjected to the laser shock wave pressure transmitted indirectly by the rubber and the shearing action of the mold. Then, plastic deformation and fracture occur, and the punched parts are formed. The experimental parameters used in the laser dynamic flexible micropunching experiment are listed in Table 1.

In the experiment, Spitlight2000, which is an Nd:YAG laser (INNOLAS Laser Corporation in Munich, Germany), was employed, operating in Gaussian distribution. The main parameters of the laser are presented in Table 2.

For the experiment preparation, polymethyl methacrylate (PMMA) was used as the blank holder, and black paint sprayed uniformly on the blank holder in advance was used as the ablative medium for generating the plasma and shock waves under laser shocking. The black paint can increase the laser energy absorption rate and prevent the rubber from being directly irradiated by the laser beam [25]. The material of the rubber used in the experiment was polyurethane, which has been proven to be favorable for plastic deformation and can homogenize energy and pressurization [26].

During the LPS process, as shown in Figure 3a, copper foil was laser pre-shocked under a laser energy density of 14.2 J/cm^2^, and the rubber, with a thickness of 100 µm, was used mainly to equalize energy and prevent the black paint from sticking directly to the surface of the workpiece [27]. Moreover, the surface roughness of a single workpiece was measured with a KEYENCE VK-X200 3D Laser Scanning Microscope (KEYENCE Corporation in Osaka, Japan). After laser dynamic flexible punching, the cross-section characteristics of the punched parts and the characteristic size of the punched holes were observed through a KEYENCE VHX-1000C digital microscope (KEYENCE Corporation in Osaka, Japan). In addition, fracture morphology was observed through a S-3400 scanning electron microscope (SEM; HITACHI Corporation in Tokyo, Japan).

### 2.2. Material Preparation

**Figure 4:**
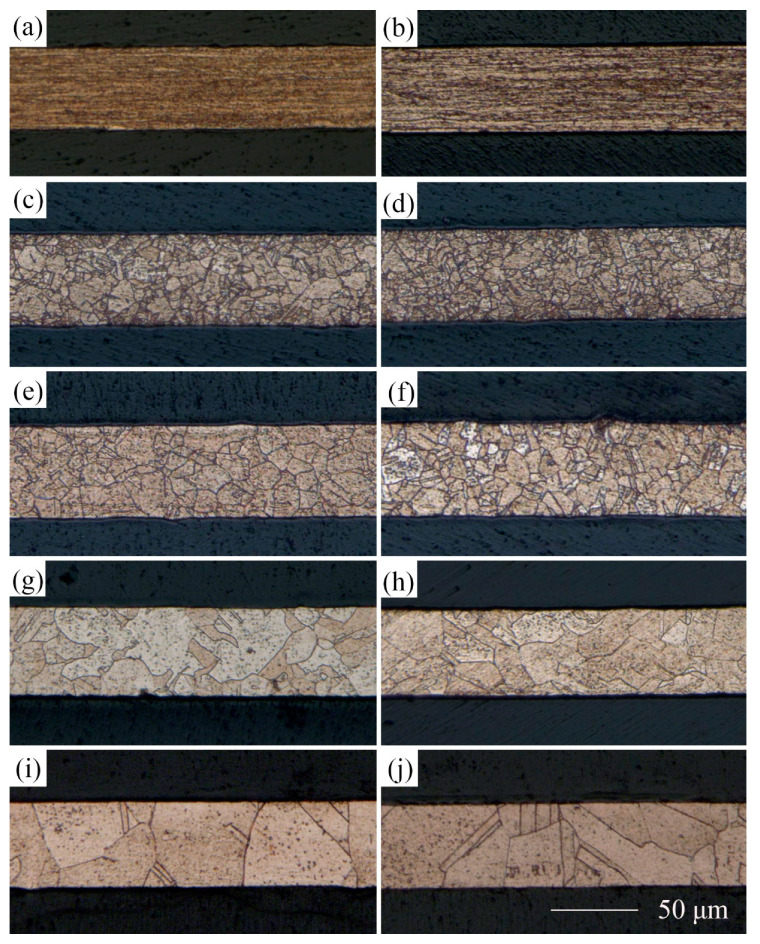
Microstructures of the copper foils with and without LPS under different annealing treatment. (**a**) without LPS, cold-rolled; (**b**) with LPS, cold-rolled; (**c**) without LPS, 350 °C, 1 h; (**d**) with LPS, 350 °C, 1 h; (**e**) without LPS, 450 °C, 1 h; (**f**) with LPS, 450 °C, 1 h; (**g**) without LPS, 550 °C, 1 h; (**h**) with LPS, 550 °C, 1 h; (**i**) without LPS, 650 °C, 1 h; (**j**) with LPS, 650 °C, 1 h.

Owing to extensive application in MEMS, the material selected for the experimental research was T2 copper foil with a thickness of 50 µm. Cold-rolled copper foils were annealed at 350, 450, 550, and 650 °C in a vacuum and held for an hour to obtain different grain sizes. The copper foils before and after LPS were etched with a solution of 5 g FeCl_3_, 15 mL HCl, and 85 mL H_2_O for 2 to 3 s to observe grain structures. Grain sizes were measured using a digital microscope (KEYENCE VHX-1000C). The metallographic structures of the different copper foils are shown in Figure 4.

## 3. Results and Discussion

### 3.1. Effect of LPS on Grain Size and Surface Roughness

The linear intercept method was used to calculate grain sizes, five fields were selected, and multiple measurements were taken to obtain the average grain size. The results are shown in Table 3. In addition, the results similar to the previous work [19,21] show that the values of average grain size of copper foil with different annealing treatment decreased by 12.2%, 19.4%, 16.3%, and 17.7% after LPS. The relationship between the thickness and grain size of the copper foils was characterized by the value of *N*, which is the ratio of the thickness t to grain size d (N=t/d). The results are shown in Table 4.

**Figure 5:**
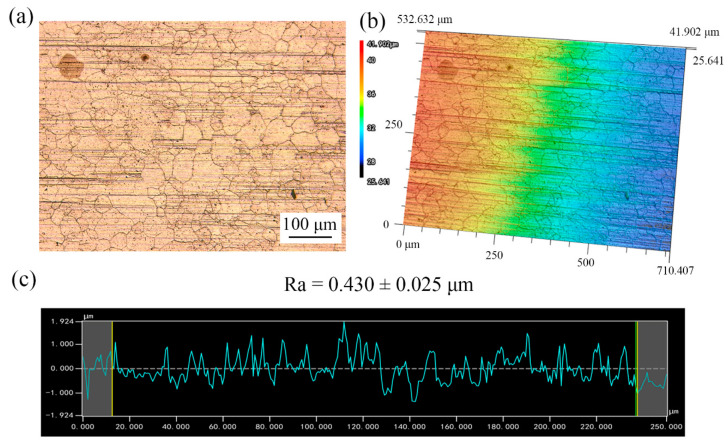
Surface topography of copper foils annealed at 650 °C without LPS. (**a**) 2D surface topography; (**b**) 3D surface topography; (**c**) roughness curve.

**Figure 6:**
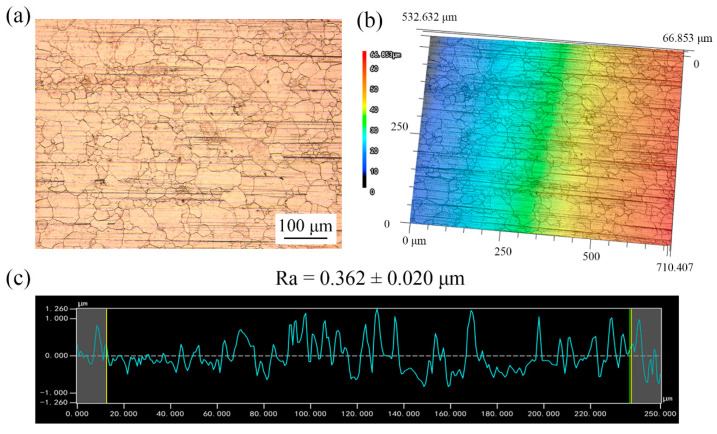
Surface topography of copper foils annealed at 650 °C with LPS. (**a**) 2D surface topography; (**b**) 3D surface topography; (**c**) roughness curve.

Figure 5 and Figure 6 show the surface topography of the copper foils without and with LPS. Compared with Figure 5a, plenty of fine grains appear around the large grains in Figure 6a, indicating that the grain refinement happens after LPS. The surface roughness is an average value of surface height of all the statistical points; each workpiece was selected five regions to be tested for calculating the average value of $R_{a}$. The surface roughness $R_{a}$ of copper foil annealed at 650 °C decreases from 0.430 to 0.362 µm after LPS (shown in Figure 5c and Figure 6c). In addition, the surface height varies from −1.924 to 1.924 µm, while it varies from −1.260 to 1.260 µm after LPS. This indicates that the LPS could improve the surface quality.

### 3.2. Fracture Morphology

**Figure 7:**
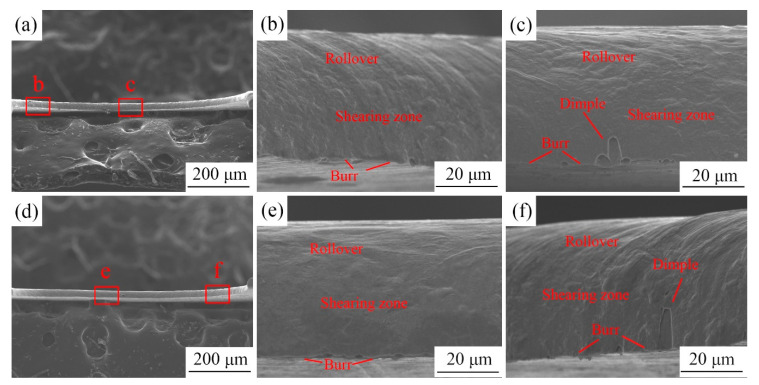
Comparison of the fracture surface of punched parts under a laser energy density of 28.3 J/cm^2^. (**a**) The punched part without LPS; (**b**) and (**c**) respectively correspond to the regions “b”, “c” in (**a**); (**d**) the punched part with LPS; (**e**) and (**f**) respectively correspond to the regions “e”, “f” in (**d**).

**Figure 8:**
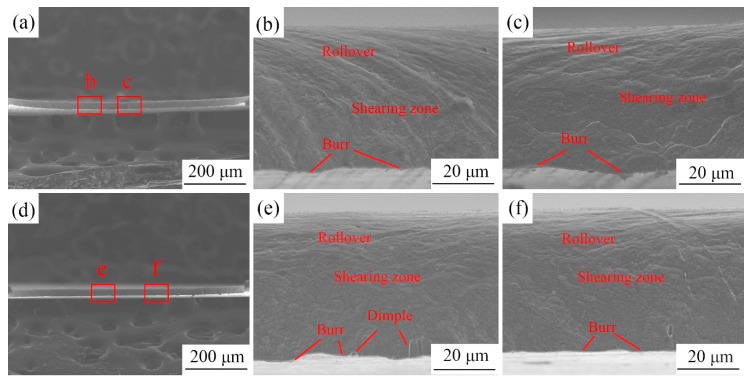
Comparison of the fracture surface of punched parts under a laser energy density of 38.2 J/cm^2^. (**a**) The punched part without LPS; (**b**) and (**c**) respectively correspond to the regions “b”, “c” in (**a**); (**d**) the punched part with LPS; (**e**) and (**f**) respectively correspond to the regions “e”, “f” in (**d**).

To illustrate the effect of LPS on the morphology of the copper foils after laser dynamic flexible micropunching, the fracture morphology of the cold-rolled copper foils punched under a laser energy power density of 28.3 and 38.2 J/cm^2^ is shown in Figure 7 and Figure 8, respectively. In contrast to fracture surface morphology under quasistatic loading conditions, the fracture surface morphology under dynamic high strain rate loading conditions has no fracture zone, which is in line with the results of Zheng et al. [17]. In addition, the fracture surface can be divided into three regions, namely, the rollover, shearing zone, and burr. The fracture mode of the punched parts is a typical ductile fracture. Based on the observations in Figure 7c,f, the fracture surface of the punched parts with LPS has larger dimples than those without LPS, thereby indicating that LPS improves the plasticity of the foils. The disappearance of the fracture zone during the laser dynamic flexible micropunching process can be attributed to the following reasons. (1) Laser dynamic flexible micropunching belongs to high strain rate forming, and the rubber homogenizes energy and pressurization, thereby extending the laser shocking time [26]. Moreover, the severe plastic deformation and short deformation time make the whole fracture surface present a kind of ductile fracture mode. (2) The pressure of the laser shock wave is too large, reaching the order of GPa, thereby generating excessive compressive stress in the deformation region. (3) The laser beam diameter (2 mm) is larger than the mold diameter (1.5 mm). Thus, the deformation region is larger than the diameter of the mold hole. The negative clearance makes it possible to enhance compressive stress during the shear separation process.

### 3.3. Section Morphology

**Figure 9:**
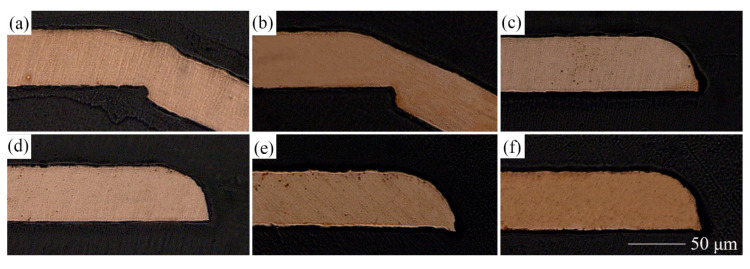
Section morphology of punched parts without LPS under different laser power densities. (**a**) 10.3; (**b**) 11.6; (**c**) 14.2; (**d**) 18.3; (**e**) 28.3; (**f**) 38.2 J/cm^2^.

**Figure 10:**
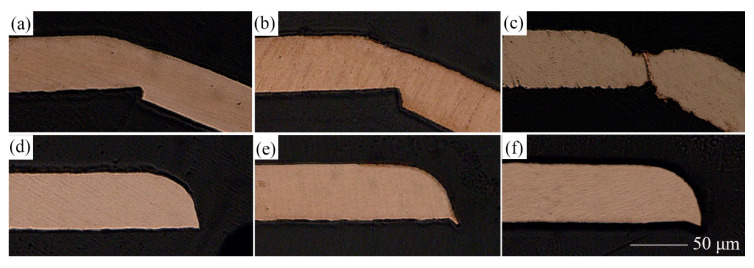
Section morphology of punched parts with LPS under different laser power densities. (**a**) 10.3; (**b**) 11.6; (**c**) 14.2; (**d**) 18.3; (**e**) 28.3; (**f**) 38.2 J/cm^2^.

The effect of LPS on the section morphology of the cold-rolled copper foils after laser dynamic flexible micropunching is shown in Figure 9 and Figure 10. The grains of the cold-rolled copper foils are too slender. Thus, measuring grain size accurately is difficult. Figure 3 shows that the grains of the copper foils are refined significantly after LPS, which corresponds to the data in Table 3, thereby suggesting that grain refinement also occurs in the cold-rolled copper foils. Combining Figure 9c and Figure 10c, the copper foils with LPS are not completely broken, whereas those without LPS are broken after laser dynamic flexible micropunching under a laser energy density of 14.2 J/cm^2^. These findings indicate that LPS increases the tensile strength of the workpiece. By combining Figure 9d–f and Figure 10d–f, it can be observed that the punched copper foils with LPS have slenderer burrs than those without LPS.

**Figure 11:**
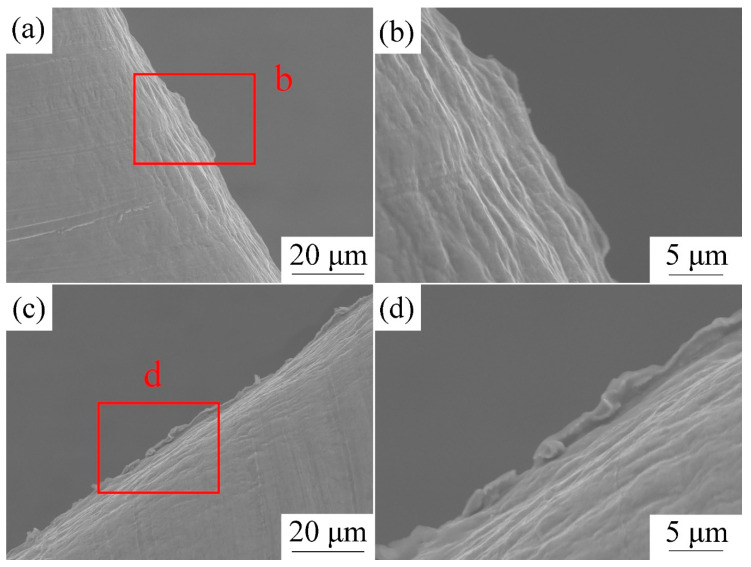
SEM observations of the punched holes under a laser energy density of 38.2 J/cm^2^. (**a**) The punched hole without LPS; (**b**) corresponds to the region “b” in (**a**); (**c**) the punched hole with LPS; (**d**) corresponds to the region “d” in (**c**).

Figure 11a,c show SEM observations of an image of the punched holes of the cold-rolled copped foils with and without LPS. From the surface morphology comparison, increased burrs on the surface of the punched holes with LPS are noted. Furthermore, a small rollover zone is observed on the surface of the punched holes with LPS in Figure 11b,c. To further explore the effect of LPS on the fracture surface, with reference to Meng et al. [28], an analysis of the depth of different regions of the fracture surface is performed. The comparison of the section morphology of the punched copper foils in Figure 9 and Figure 10 shows that the burr can be identified easily, whereas defining the boundary between the shearing zone and rollover is difficult. The shearing zone is mostly inclined. Thus, we set the standard when the angle between the fracture surface and punching direction is less than 10°, as shown in Figure 9.

**Figure 12:**
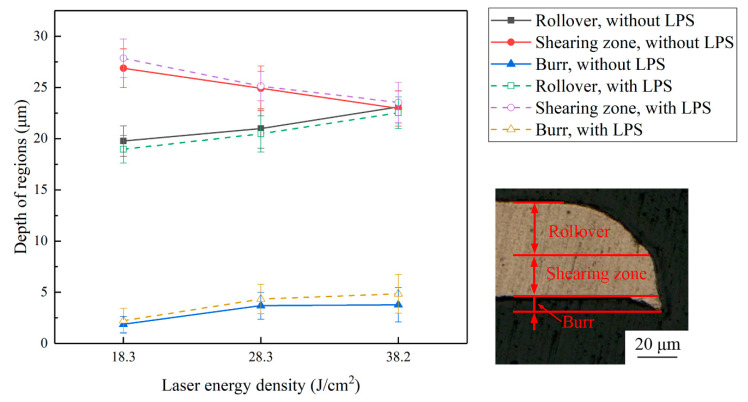
Influence of the laser energy density on the depth of different regions of punched parts with and without LPS.

Figure 12 shows the depth of each region of the fracture surface of the cold-rolled copper foils under a laser energy power density of 18.3, 28.3, and 38.2 J/cm^2^. An interesting phenomenon is that the rollover and burr increase with the laser energy density increasing and the shearing zone decreasing simultaneously, which suggests that the forming quality in terms of the fracture morphology deteriorates. Moreover, the burr of the fracture surface of the copper foils with LPS after laser dynamic flexible micropunching is longer than that of the fracture surface of the copper foils without LPS. The trend of the rollover is completely opposite to that of the burr, and the fracture surface of the copper foils with LPS has a larger shearing zone, which means that the forming quality of the copper foils with LPS demonstrates a certain degree of improvement.

**Figure 13:**
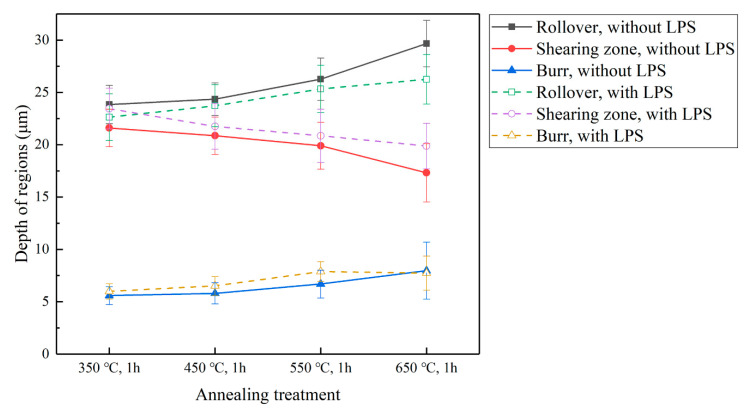
Influence of the initial grain size on the depth of different regions of punched parts with and without LPS under a laser energy density of 38.2 J/cm^2^.

As shown in Figure 13, under the same laser energy density, grain size also has an effect on fracture surface morphology. An increase in grain size (a decrease in the N value) results in an increase in the rollover and burr and a decrease in the shearing zone, whereas for the copper foil annealed at 650 °C, the change trend of each region becomes abnormal. In addition, the effect of LPS on the fracture surface of the copper foils with different annealing treatments is superior to that on the fracture surface of the copper foils under different laser power densities. Therefore, the LPS makes increased shearing zone and decreased rollover on the fracture surface to obtain better forming quality.

The phenomenon of a large rollover for all the workpieces exists, which varies from 18.9 to 23.1 µm in Figure 12 and from 22.6 to 29.7 µm in Figure 13. This phenomenon may be attributed to the following reasons. The laser spot is larger than the characteristic aperture of the punching mold, so that the pressure of the laser shock wave affects the entire mold hole and is applied to the outside of the mold hole. Thus, bending deformation occurs preferentially on the upper surface of the workpiece, and the upper side is pulled into the die hole along the punching direction, thereby causing the bottom surface of the workpiece to begin extrusion molding. During this process, the rubber is tightly attached to the upper surface of the workpiece. Because the thickness of rubber (300 µm) is larger than the thickness of the workpiece (50 µm), the rollover caused by the bending deformation will be larger. According to the surface layer model, in the surface layer, the bending deformation occurs easily, owing to the reduced constraining force of individual grains. Thus, the flow stress on the surface layer is smaller than that in the interior regions [29]. Meanwhile, as high strain rate sensitivity and the inertial effect are favorable for delaying void growth [30], the forming capability of the workpiece improves, and fracture failure is less likely to occur, thereby extending bending time and resulting in a large rollover on the fracture surface. With the increase in the laser energy density, the severe bending deformation results in larger rollover. As grain size increases, the flow stress of the workpiece decreases so that the plastic deformation more easily happens [31]. Hence, the larger grain results in larger rollover on the fracture surface.

### 3.4. Shape Accuracy and Dimensional Accuracy

In this section, to further illustrate the effect of LPS on the forming quality of the punched parts during laser dynamic flexible micropunching, the shape accuracy and dimensional accuracy of the punched holes are characterized.

#### 3.4.1. Dimensional Accuracy

**Figure 14:**
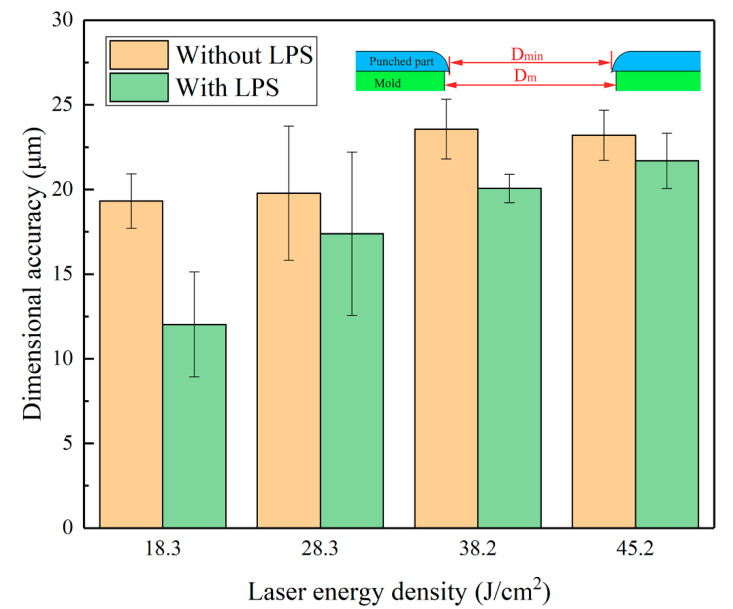
Influence of the laser energy density on the dimensional accuracy of punched holes with LPS and without LPS.

To further study the forming quality of the copper foils in laser dynamic flexible micropunching, $D_{a}$ is defined as the dimensional accuracy, as follows:

(1) $$D_{a}=D_{m}-D_{min}$$

where $D_{m}$ is the characteristic aperture of the punching mold, $D_{min}$ is the minimum aperture diameter of the bottom surface of the punched part, and the measurement method is shown in Figure 14. Each punched hole was measured three times to calculate the average value, and five workpieces were tested under each condition to calculate the standard deviation. The diameter of the punched hole is close to the diameter of the mold hole as the $D_{a}$ decreases, thereby resulting in improved dimensional accuracy.

**Figure 15:**
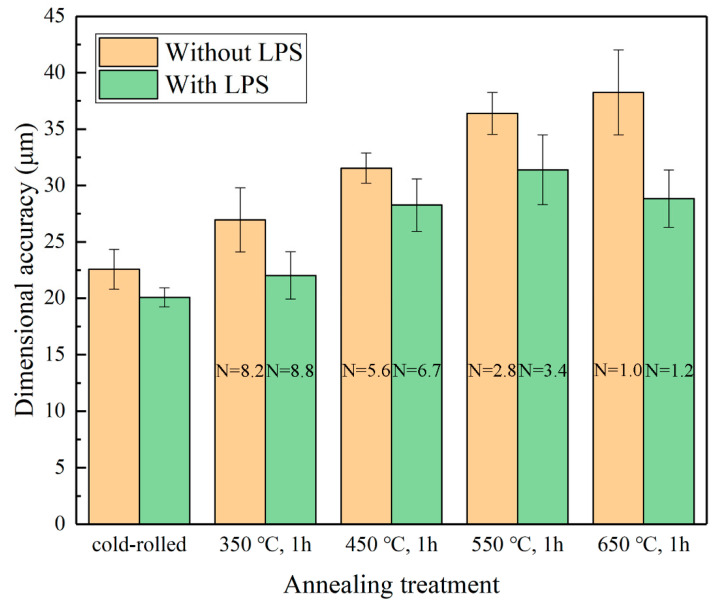
Influence of the initial grain size on the dimensional accuracy of punched holes with LPS and without LPS under a laser energy density of 38.2 J/cm^2^.

Figure 14 and Figure 15 demonstrate the influence of LPS on the dimensional accuracy of the punched holes of the cold-rolled copper foils. As laser energy density increases, the $D_{a}$ increases, as shown in Figure 14. However, the change of the $D_{a}$ caused by the increase in the laser energy density is distributed between 19.3 and 23.6 µm. The results mean that the increase in the laser energy density cannot effectively improve the dimensional accuracy. Meanwhile, the $D_{a}$ of the punched holes of the copper foils with LPS is smaller than that of the punched holes of the copper foils without LPS, thereby resulting in better dimensional accuracy. Specifically, Figure 14 shows that the dimensional accuracy improvement caused by LPS is the most significant under a laser energy density of 18.3 J/cm^2^.

As shown in Figure 15, under the same laser energy power density condition, as grain size increases (the N value decreases), the $D_{a}$ also becomes larger, thereby decreasing dimensional accuracy accordingly. Moreover, compared with the punched holes of the copper foils without LPS, the punched holes of the copper foils with LPS can obtain better dimensional accuracy. When grain size is close to the thickness of the copper foil (N≈1), the change trend of dimensional accuracy becomes abnormal. In reality, the values of $D_{a}$ decrease significantly, which are expected to increase theoretically, because the mechanical properties of a single grain have a significant impact in this case.

**Figure 16:**
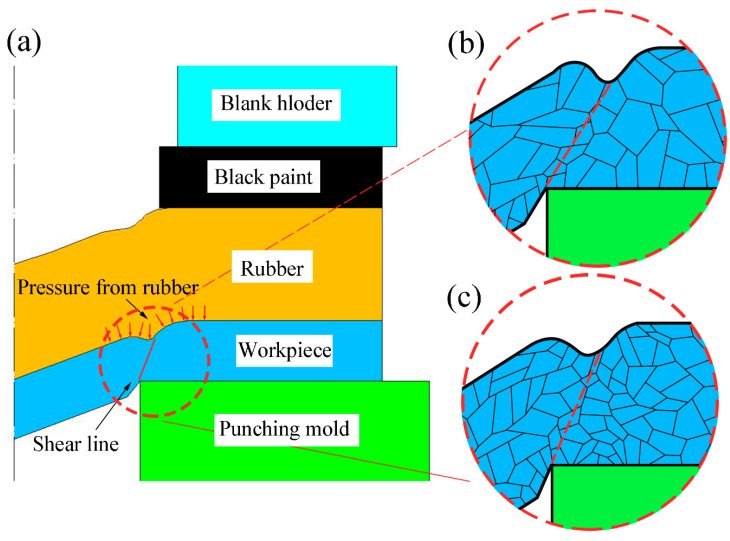
Deformation behavior of the workpiece during laser dynamic flexible micropunching and the influence of LPS on the dimensional accuracy of punched holes. (**a**) Schematic of deformation behavior; (**b**) copper foil without LPS; (**c**) copper foil with LPS.

Figure 16 presents the deformation behavior of the workpiece during laser dynamic flexible micropunching and the effect of LPS on the dimensional accuracy of punched holes. As shown in Figure 16a, the rubber is tightly attached to the upper surface of the workpiece during the deformation process. It can be understood that the pressure from the rubber is always along the radial direction of the deformed surface. As the crack extends, the internal grains are sheared apart with the direction of plastic deformation and the shear line will shift towards the entrance of the mold under the pressure from rubber. The surface roughening phenomenon in plastic deformation causes uneven deformation and further affects the fracture strain. The rate of increase in the surface roughness to thickness increased with the decrease in the number of grains in the thickness direction, which leads to low fracture strain [32,33]. It means that the copper foil with LPS has higher fracture strain, so that the shear line is closer to the mold entrance (shown in Figure 16b,c). Therefore, the value of the $D_{min}$ of punched holes with LPS will be larger, and the $D_{a}$ will increase accordingly. Thus, the dimensional accuracy of punched holes with LPS will be improved.

#### 3.4.2. Shape Accuracy

**Figure 17:**
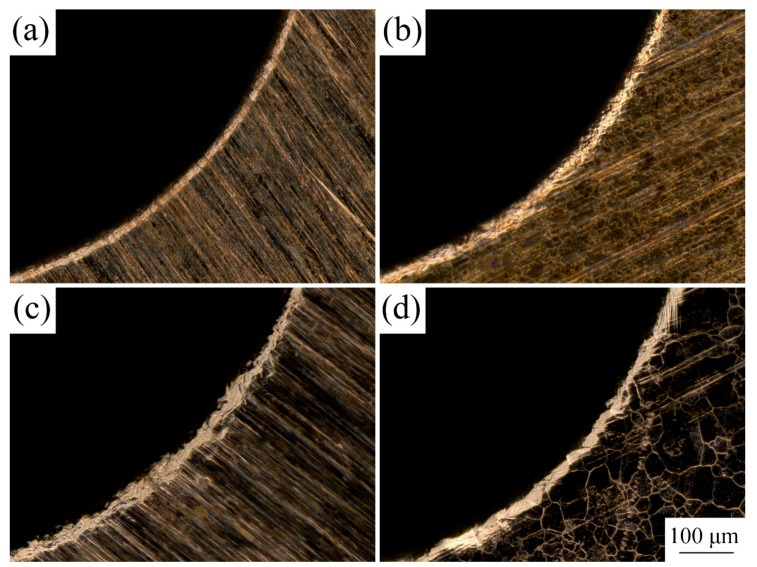
Comparison of punched holes without LPS under different conditions (upper surface). (**a**) Cold-rolled; (**b**) 450 °C annealed, 1 h; (**c**) 550 °C annealed, 1 h; (**d**) 650 °C annealed, 1 h.

**Figure 18:**
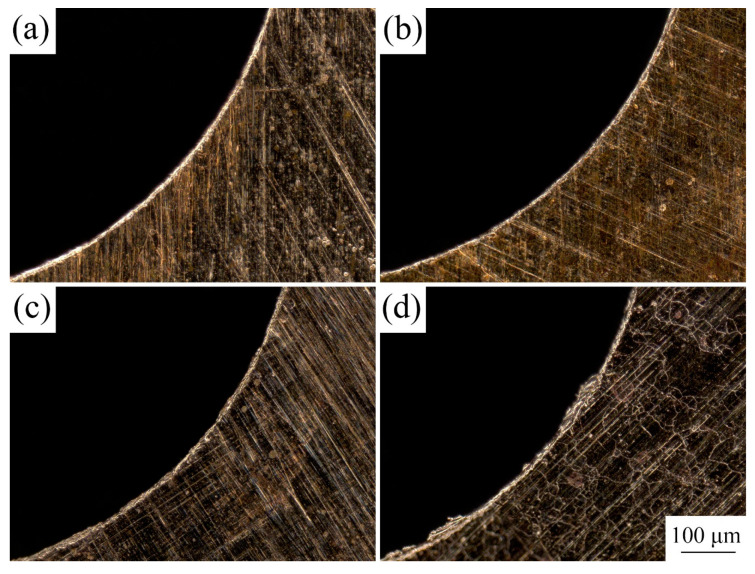
Comparison of punched holes without LPS under different conditions (bottom surface). (**a**) Cold-rolled; (**b**) 450 °C annealed, 1 h; (**c**) 550 °C annealed, 1 h; (**d**) 650 °C annealed, 1 h.

Figure 17 and Figure 18 show that the punched holes of the copper foils without LPS are cold-rolled and annealed at 450, 550, and 650 °C. In this laser dynamic flexible punching experiment, laser energy density was set to 38.2 J/cm^2^, and the upper surface in Figure 17 refers to the surface facing the laser shock wave. In addition, the bottom surface in Figure 18 refers to the surface in contact with the punching mold. The copper foils are broken completely under this laser energy density, which indicates that the shock wave caused by the laser pulse exceeds the maximum shear strength of the workpiece.

**Figure 19:**
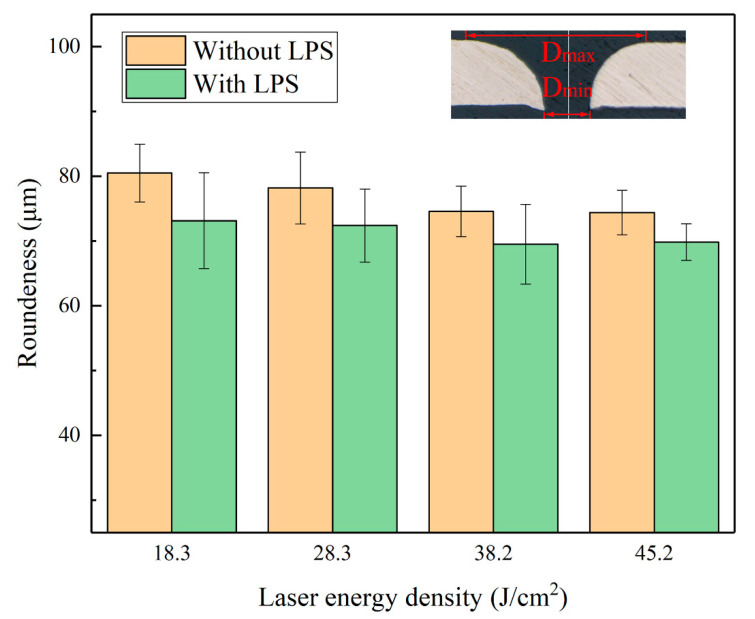
Influence of the laser energy density on the shape accuracy of punched holes with and without LPS.

Corresponding to the data in Table 4, the contours of the punched holes with fine grain sizes are smooth (Figure 17a,b and Figure 18a,b), while those with coarse grains sizes are irregular (Figure 17c,d and Figure 18c,d). To characterize the roundness error and shape accuracy of the punched holes and analyze how LPS impacts shape accuracy, Roundness is defined to characterize the shape accuracy of the punched holes based on Joo et al. [4], as follows:

(2) $$Roundness=D_{max}-D_{min}$$

where $D_{max}$ is the maximum hole diameter on the upper surface of the punched parts and $D_{min}$ is the minimum hole diameter on the bottom surface of the punched parts. The basic measurement method of Roundness is shown in Figure 19. The smaller the Roundness, the higher the shape accuracy of the punched hole.

Figure 19 presents the Roundness results of the punched holes of cold-rolled copper foils with and without LPS punched under different laser energy density conditions. It can be found that laser energy density has little effect on the shape accuracy of punched holes, because the average values of Roundness of punched holes without LPS vary from 74.4 to 80.5 µm, and those with LPS vary from 69.5 to 73.2 µm. Meanwhile, under the same laser energy density condition, the values of Roundness of punched holes with LPS are further reduced, thereby indicating that LPS is beneficial to obtain improved shape accuracy. This finding may be because LPS refines the internal grains of copper foils, as shown in Figure 3.

**Figure 20:**
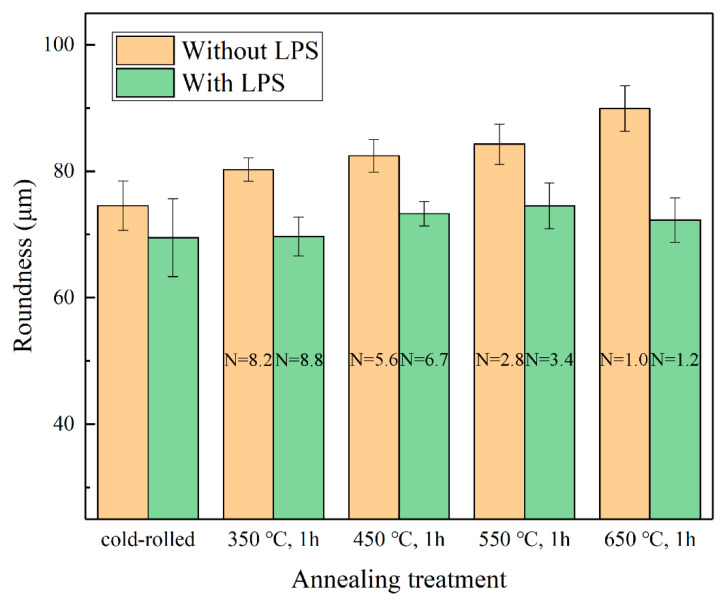
Influence of initial grain size on the shape accuracy of punched holes with and without LPS under a laser energy density of 38.2 J/cm^2^.

**Figure 21:**
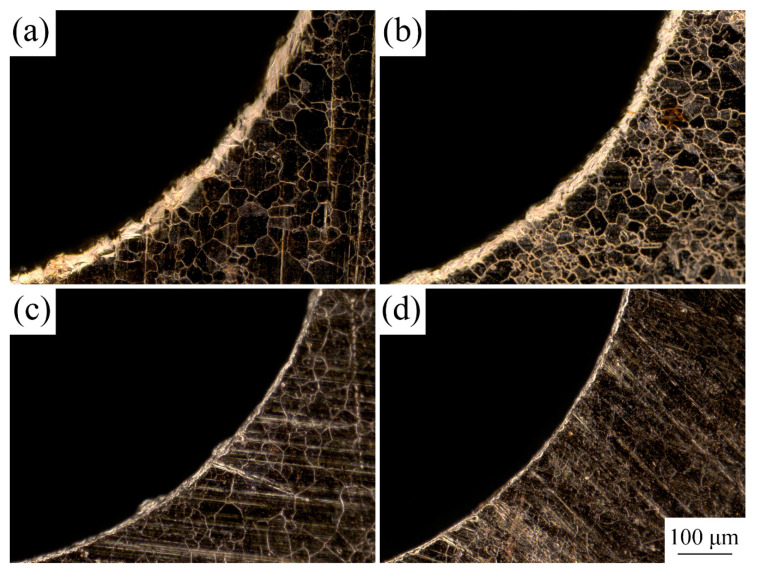
Influence of LPS on the shape accuracy of punched holes of copper foils annealed at 650 °C under a laser energy density of 38.2 J/cm^2^. (**a**) Upper surface without LPS; (**b**) upper surface with LPS; (**c**) bottom surface without LPS; (**d**) bottom surface with LPS.

Figure 20 presents the Roundness results of the punched holes of the copper foils with different annealing treatment states under a laser energy density of 38.2 J/cm^2^. The results suggest that as grain size increases (the N value decreases), the values of Roundness increase and shape accuracy deteriorates. Moreover, the values of Roundness of punched holes with LPS all reduced compared with those without LPS. The grain refinement after LPS can be easily found, and the contours of the punched holes are obviously smoother (shown in Figure 21a,b). It is worth noting that the change trend of Roundness becomes abnormal for the punched holes of copper foils annealed at 650 °C.

**Figure 22:**
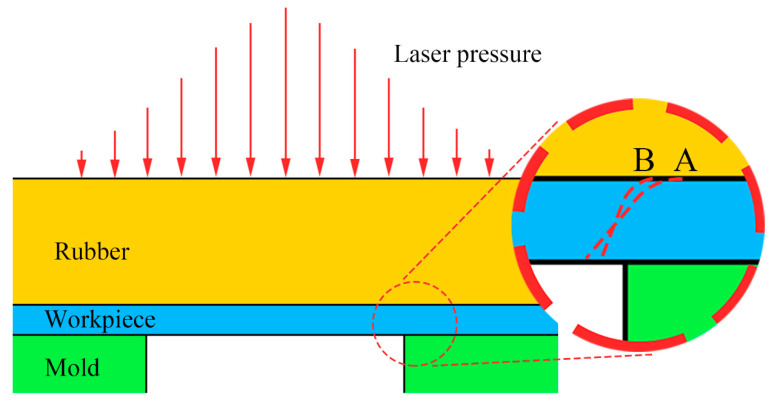
Force acting before laser dynamic flexible micropunching.

As shown in Figure 22, since the laser energy is operating in Gaussian distribution, the closer to the edge of the laser spot, the smaller the pressure on the rubber and workpiece. When the pressure is higher than the yield strength of the workpiece, the workpiece begins plastic deformation. It is assumed that the section profile of the fracture surface without LPS follows curve A in Figure 22. Since the surface grains after LPS are refined, the yield strength and flow stress increase [32]. Thus, compared with the punched holes without LPS, the value of $D_{max}$ should be reduced. Meanwhile, the increase in the number of grains in the thickness direction caused by LPS results in an increase in the fracture strain, so that the value of $D_{min}$ will be increased. Thus, the section profile of the fracture surface with LPS is supposed to follow curve B. Therefore, the LPS leads to an increase in the value of Roundness, indicating that the shape accuracy will be increased.

In summary, given that an increase in laser energy power density has little effect on the shape accuracy and dimensional accuracy of the punched holes, an increase in grain size results in poor shape accuracy and dimensional. LPS can be performed to improve the laser dynamic flexible micropunching process, because LPS can generally improve the dimensional accuracy, and especially for the copper foil with coarse grains, the improvement is particularly significant.

**Figure 23:**
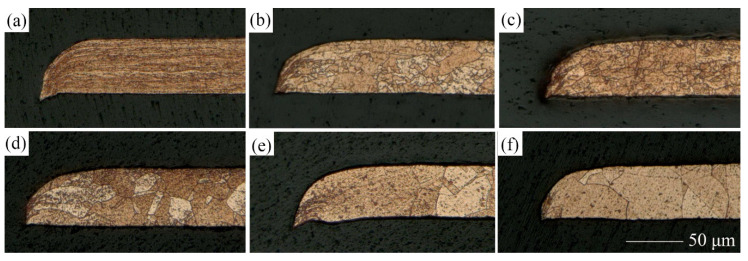
Microstructures of the punched parts without LPS under a laser energy density of 38.2 J/cm^2^. (**a**) Cold-rolled; (**b**) 350 °C annealed, 1 h; (**c**) 450 °C annealed, 1 h; (**d**) 550 °C annealed, 1 h; (**e**) 650 °C annealed, 1 h; (**d**) 650 °C annealed, 1 h.

**Figure 24:**
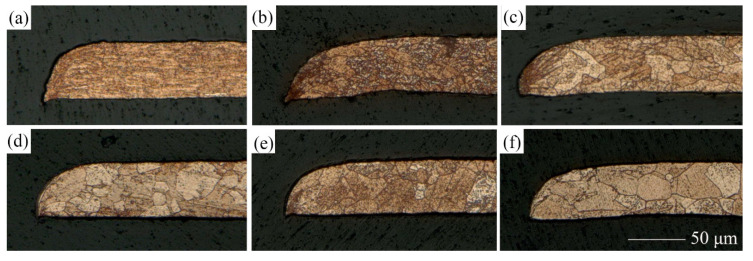
Microstructures of the punched parts with LPS under a laser energy density of 38.2 J/cm^2^. (**a**) Cold-rolled; (**b**) 350 °C annealed, 1 h; (**c**) 450 °C annealed, 1 h; (**d**) 550 °C annealed, 1 h; (**e**) 650 °C annealed, 1 h; (**d**) 650 °C annealed, 1 h.

To further investigate the effect of LPS on the shape accuracy and dimensional accuracy of punched holes, the obtained punched parts were etched, and the microstructures are shown Figure 19 and Figure 20. Referring to the data in Table 4, when t >d (e.g., N = 8.2, 5.9, 2.8 in Figure 23 and N = 8.8, 6.7, 3.4 in Figure 24), laser dynamic flexible micropunching is a polycrystalline deformation owing to multiple grains in the punching direction. As grain size increases (the N value decreases), indicating that the number of grains in the punching direction increases, the flow stress of the workpiece gradually increases [31,34]. Thus, the depth of the rollover decreases accordingly.

When t≈d (e.g., N = 1.0 in Figure 23 and N = 1.2 in Figure 24), the mechanical properties of a single grain are extremely important, because grain orientation has a substantial impact on the shape accuracy and dimensional accuracy of the punched holes. The Roundness and $D_{a}$ of punched holes annealed at 650 °C show an abnormal trend in Figure 15 and Figure 20. At N = 1.0, the Roundness and the $D_{a}$ increase significantly, whereas at N = 1.2, the Roundness and the $D_{a}$ decrease instead. This finding means that as grain size is close to the foil thickness (N = 1.0, 1.2), predicting the change trend becomes extremely difficult. Combined with the work of Xu et al. [35], the number and distribution of grains in the thickness direction of the copper foils are important factors that affect deformation behavior during laser dynamic flexible micropunching. This finding is because shear deformation is concentrated on several grains between the laser spot and the feature hole of the punching mold. Although laser dynamic flexible micropunching is a type of high strain rate deformation, grain size still plays an important role in the plastic deformation of the copper foils. Specifically, when grain size is close to the thickness of the copper foil (N≈1), grain orientation affects the plastic deformation significantly. When the grain is deformed in a given applied stress direction, the best sliding direction exists, leading to the largest shear stress [36]. If the grain sliding direction is not consistent with the laser dynamic flexible micropunching direction, then, the plastic deformation will be nonuniform, thereby resulting in poor shape accuracy and dimensional accuracy. This finding may be the reason for the poor forming quality of the punched holes without LPS annealed at 650 °C (shown in Figure 23e). By contrast, the shape accuracy and dimensional accuracy of the punched holes with LPS annealed at 650 °C may be fine (shown in Figure 23f), because the grain sliding direction that is consistent with the laser dynamic flexible micropunching direction is conductive to plastic deformation.

As shown in Figure 23e, grain orientation is not conducive to the laser dynamic flexible micropunching direction. Instead, the yield stress increases with large deviation as the grain size increases [34], and the bending deformation is severe, thereby reducing the shape accuracy and dimensional accuracy of the punched holes. Meanwhile, as shown in Figure 23f, grain orientation is favorable to the laser dynamic flexible micropunching direction, thereby increasing the shape accuracy and dimensional accuracy of the punched holes instead. Thus, whether the laser dynamic flexible micropunching process is a polycrystalline deformation or a single crystal deformation depends on the number of grains in the punching direction. Thus, with reference to Zheng et al. [17], the difference in grain orientation will cause unpredictable forming quality of punched parts. In this case, as shown in Figure 23e,f, the section profiles of fracture surface with LPS are similar to the work of Zheng et al. [17], but the number of grains in the thickness direction has been increased under the action of LPS, so that the punching process can be promised to be a polycrystalline deformation and the forming quality can be improved, as shown in Figure 24e,f. The LPS can be applied to reduce the possibility of single crystal deformation during laser dynamic flexible micropunching, thereby ensuring the stability of the dynamic flexible micropunching process.

## 4. Conclusions

Experimental research on the effect of LPS on the forming quality of punched parts after laser dynamic flexible micropunching was conducted in this study. The punched parts of the copper foils with LPS were compared with those without LPS in terms of the depth of different regions on the fracture surface, the shape accuracy and dimensional accuracy of the punched holes, and microstructure. The following conclusions can be drawn.

1. Grains are refined, and the surface roughness of copper foil decreases after LPS. For the copper foils annealed at 650 °C, the average grain size decreases from 51.9 to 42.7 µm, and the surface roughness $R_{a}$ decreases from 0.430 to 0.362 µm after LPS.
2. The increase in laser energy density and grain size lead to the deterioration of the fracture surface. LPS can improve the quality of the fracture surface because the increase in the flow stress and yield caused by LPS result in an increase in the shearing zone and a decrease in the rollover.
3. The dimensional and shape accuracy of punched holes can be improved by LPS compared with punched holes without LPS, because the LPS suppresses surface roughening in plastic deformation and improves fracture strain.
4. When the grain size is close to the thickness of the copper foil, the forming quality of the punched parts becomes uncertain, because the mechanical properties of a single grain cannot be ignored in this case. LPS can reduce the uncertainty of laser dynamic flexible micropunching to improve the stability of the laser dynamic flexible micropunching process.

## Figures and Tables

**Table 1:** Detailed experimental parameters.

Parameters	Values
Laser beam diameter (mm)	2
Mold hole diameter (mm)	1.5
PMMA thickness (mm)	3
Black paint thickness (µm)	~50
Rubber thickness used in LPS (µm)	100
Rubber thickness used in the micropunching (µm)	300
Laser energy density (J/cm^2^)	10.3, 11.6, 14.2, 18.3, 28.3, 38.2, 45.2

**Table 2:** Main parameters of the Spitlight2000, an Nd: YAG Laser.

Laser Parameters	Values
Laser energy (mJ)	80–1800
Pulse width (ns)	8
Wave length (nm)	1064
Exit spot diameter (mm)	9

**Table 3:** Average grain sizes of the copper foils under different conditions.

Annealing Treatment	350 °C, 1 h	450 °C, 1 h	550 °C, 1 h	650 °C, 1 h
Without LPS (µm)	7 ± 3	10 ± 4	19 ± 6	52 ± 6
With LPS (µm)	6 ± 2	8 ± 3	16 ± 5	42 ± 6

**Table 4:** Values of the ratio of average grain size to thickness (the N value) of the copper foils under different conditions.

Annealing Treatment	350 °C, 1 h	450 °C, 1 h	550 °C, 1 h	650 °C, 1 h
Without LPS	8.2 ± 4.1	5.6 ± 2.0	2.8 ± 0.8	1.0 ± 0.1
With LPS	8.8 ± 3.1	6.7 ± 2.2	3.4 ± 1.0	1.2 ± 0.2

## References

[1] Teyfouri A., Ahmadi M., Lori E.S., Sorooshian S. (2015). A Review on Micro Formings. Mod. Appl. Sci..

[2] Saotome Y., Yasuda K., Kaga H. (2001). Microdeep drawability of very thin sheet steels. J. Mater. Process. Technol..

[3] Gau J.T., Principe C., Yu M. (2007). Springback behavior of brass in micro sheet forming. J. Mater. Process. Technol..

[4] Joo B.-Y., Rhim S.-H., Oh S.-I. (2005). Micro-hole fabrication by mechanical punching process. J. Mater. Process. Technol..

[5] Wielage H., Vollertsen F. (2011). Classification of laser shock forming within the field of high speed forming processes. J. Mater. Process. Technol..

[6] Liu H., Shen Z., Wang X., Wang H., Tao M. (2010). Numerical simulation and experimentation of a novel micro scale laser high speed punching. Int. J. Mach. Tools Manuf..

[7] Wang X., Qiu T., Shen Z., Zhang D., Ma Y., Gu Y., Liu H. (2016). Forming Properties of a Microscale Laser Dynamic Flexible Forming Technique. Mater. Manuf. Process..

[8] Liu H., Lu M., Wang X., Shen Z., Gu C., Gu Y. (2013). Micro-punching of aluminum foil by laser dynamic flexible punching process. Int. J. Mater. Form..

[9] Wang X., Qian Q., Shen Z., Li J., Zhang H., Liu H. (2015). Numerical simulation of flexible micro-bending processes with consideration of grain structure. Comput. Mater. Sci..

[10] Shen Z., Zhang J., Liu H., Wang X., Ma Y. (2019). Reducing the rebound effect in micro-scale laser dynamic flexible forming through using plasticine as pressure-carrying medium. Int. J. Mach. Tools Manuf..

[11] Zhang K.F., Kun L. (2009). Classification of size effects and similarity evaluating method in micro forming. J. Mater. Process. Technol..

[12] Xu J., Li J., Shi L., Shan D., Guo B. (2015). Effects of temperature, strain rate and specimen size on the deformation behaviors at micro/meso-scale in ultrafine-grained pure Al. Mater. Charact..

[13] Janssen P.J.M., de Keijser T.H., Geers M.G.D. (2006). An experimental assessment of grain size effects in the uniaxial straining of thin Al sheet with a few grains across the thickness. Mater. Sci. Eng. A.

[14] Zheng C., Zhang X., Liu Z., Ji Z., Yu X., Song L. (2018). Investigation on initial grain size and laser power density effects in laser shock bulging of copper foil. Int. J. Adv. Manuf. Technol..

[15] Raulea L.V., Goijaerts A.M.A., Govaert L.L., Baaijens F.F. (2001). Size effects in the processing of thin metal sheets. J. Mater. Process. Technol..

[16] Fenske H., Vollertsen F. (2019). Laser shock punching: Principle and influencing factors. Prod. Eng..

[17] Zheng C., Zhang X., Zhang Y., Ji Z., Luan Y., Song L. (2018). Effects of laser power density and initial grain size in laser shock punching of pure copper foil. Opt. Lasers Eng..

[18] Shen Z., Zhang J., Li P., Liu H., Yan Z., Ma Y., Wang X. (2019). Deformation and fracture behaviors of copper sheet in laser dynamic flexible forming. J. Manuf. Process..

[19] Lu J.Z., Luo K.Y., Zhang Y.K., Sun G.F., Gu Y.Y., Zhou J.Z., Ren X.D., Zhang X.C., Zhang L.F., Chen K.M. (2010). Grain refinement mechanism of multiple laser shock processing impacts on ANSI 304 stainless steel. Acta Mater..

[20] Zhang W., Yao Y.L., Noyan I.C. (2004). Microscale Laser Shock Peening of Thin Films, Part 2: High Spatial Resolution Material Characterization. J. Manuf. Sci. Eng..

[21] Luo K.-Y., Lu J.-Z., Zhang L.-F., Zhong J.-W., Guan H.-B., Qian X.-M. (2010). The microstructural mechanism for mechanical property of LY2 aluminum alloy after laser shock processing. Mater. Des..

[22] Dai F.Z., Lu J.Z., Zhang Y.K., Luo K.Y., Wang Q.W., Zhang L., Hua X.J. (2012). Effect of initial surface topography on the surface status of LY2 aluminum alloy treated by laser shock processing. Vacuum.

[23] Haifeng Y., Fei X., Yan W., Le J., Hao L., Jingbin H. (2020). Manufacturing profile-free copper foil using laser shock flattening. Int. J. Mach. Tools Manuf..

[24] Man J., Yang H., Wang Y., Chen H., Xiong F. (2019). Study on controllable surface morphology of the micro-pattern fabricated on metallic foil by laser shock imprinting. Opt. Laser Technol..

[25] Zheng C., Sun S., Ji Z., Wang W., Liu J. (2010). Numerical simulation and experimentation of micro scale laser bulge forming. Int. J. Mach. Tools Manuf..

[26] Shen Z., Wang X., Liu H., Wang Y., Wang C. (2015). Rubber-induced uniform laser shock wave pressure for thin metal sheets microforming. Appl. Surf. Sci..

[27] Song L., Zhang X., Zhang Y., Li H., Ji Z., Zheng C. (2018). Shortening post-processing and improving forming quality of holes in laser shock punching with the aid of silicone rubber. Opt. Laser Technol..

[28] Meng B., Fu M.W., Fu C.M., Wang J.L. (2015). Multivariable analysis of micro shearing process customized for progressive forming of micro-parts. Int. J. Mech. Sci..

[29] Miyazaki S., Shibata K., Fujita H. (1979). Effect of specimen thickness on mechanical properties of polycrystalline aggregates with various grain sizes. Acta Metall..

[30] Li J., Gao H., Cheng G.J. (2010). Forming Limit and Fracture Mode of Microscale Laser Dynamic Forming. J. Manuf. Sci. Eng..

[31] Chan W.L., Fu M.W. (2011). Experimental studies and numerical modeling of the specimen and grain size effects on the flow stress of sheet metal in microforming. Mater. Sci. Eng. A.

[32] Meng B., Fu M.W. (2015). Size effect on deformation behavior and ductile fracture in microforming of pure copper sheets considering free surface roughening. Mater. Des..

[33] Furushima T., Tsunezaki H., Manabe K.-I., Alexsandrov S. (2014). Ductile fracture and free surface roughening behaviors of pure copper foils for micro/meso-scale forming. Int. J. Mach. Tools Manuf..

[34] Gau J.T., Principe C., Wang J. (2007). An experimental study on size effects on flow stress and formability of aluminm and brass for microforming. J. Mater. Process. Technol..

[35] Xu J., Guo B., Wang C., Shan D. (2012). Blanking clearance and grain size effects on micro deformation behavior and fracture in micro-blanking of brass foil. Int. J. Mach. Tools Manuf..

[36] Wang X., Ma Y., Shen Z., Gu Y., Zhang D., Qiu T., Liu H. (2015). Size effects on formability in microscale laser dynamic forming of copper foil. J. Mater. Process. Technol..

